# Targeting ACC1 in T cells ameliorates psoriatic skin inflammation

**DOI:** 10.1007/s00109-023-02349-w

**Published:** 2023-08-18

**Authors:** Yu-San Kao, Panagiota Mamareli, Ayesha Dhillon-LaBrooy, Philipp Stüve, Gloria Janet Godoy, Lis Noelia Velasquez, Verena Katharina Raker, Beate Weidenthaler-Barth, Fatima Boukhallouk, Francesca Rampoldi, Luciana Berod, Tim Sparwasser

**Affiliations:** 1 Institute of Medical Microbiology and Hygiene, University Medical Center of the Johannes Gutenberg-University Mainz, Mainz, Germany; 2 Institute of Molecular Medicine, University Medical Center of the Johannes Gutenberg-University Mainz, Mainz, Germany; 3 Research Center for Immunotherapy (FZI), University Medical Center, Johannes Gutenberg-University Mainz, Mainz, Germany; 4 Department of Dermatology, University Hospital, Westfälische Wilhelms-University Münster, Münster, Germany; 5 Department of Dermatology, University Medical Center of the Johannes Gutenberg University Mainz, Mainz, Germany; 6 Present Address: Leibniz Institute for Immunotherapy, University Regensburg, 93053 Regensburg, Germany; 7 Present Address: Chair for Immunology, University Regensburg, 93053 Regensburg, Germany; 8 Present Address: Department of Medicine, University Medical Center Hamburg-Eppendorf, Hamburg, Germany

**Keywords:** ACC1, Psoriasis, Regulatory T cells (Tregs), Skin inflammation, Fatty acid synthesis

## Abstract

**Abstract:**

Psoriasis is a chronic inflammatory skin disease driven by the IL-23/IL-17 axis. It results from excessive activation of effector T cells, including T helper (Th) and cytotoxic T (Tc) cells, and is associated with dysfunctional regulatory T cells (Tregs). Acetyl-CoA carboxylase 1 (ACC1), a rate-limiting enzyme of fatty acid synthesis (FAS), directs cell fate decisions between Th17 and Tregs and thus could be a promising therapeutic target for psoriasis treatment. Here, we demonstrate that targeting ACC1 in T cells by genetic ablation ameliorates skin inflammation in an experimental model of psoriasis by limiting Th17, Tc17, Th1, and Tc1 cells in skin lesions and increasing the frequency of effector Tregs in skin-draining lymph nodes (LNs).

****Key messages**:**

- ACC1 deficiency in T cells ameliorates psoriatic skin inflammation in mice.
- ACC1 deficiency in T cells reduces IL-17A-producing Th17/Tc17/dysfunctional Treg populations in psoriatic lesions.
- ACC1 deficiency in T cells restrains IFN-γ-producing Th1/Tc1 populations in psoriatic skin lesions and skin-draining LNs.
- ACC1 deficiency promotes activated CD44^+^CD25^+^ Tregs and effector CD62L^-^CD44^+^ Tregs under homeostasis and psoriatic conditions.

**Supplementary Information:**

The online version contains supplementary material available at 10.1007/s00109-023-02349-w.

## Introduction

Psoriasis is a common chronic inflammatory skin disease mediated by a complex interplay between the immune system and keratinocytes, which eventually results in uncontrolled proliferation and aberrant differentiation of keratinocytes [1]. Considerable studies on the signaling pathways and biological therapies targeting IL-23 and IL-17A have highlighted the IL-23/IL-17 axis as the pivotal pathogenic loop [2]. IL-17A, mainly produced by activated T cells in response to IL-23 secreted from dendritic cells, triggers keratinocyte hyperproliferation and massive immune cell recruitment [1–3]. Even though biologics including anti-IL17A, anti-IL-12p40 or IL-23p40, and anti-IL-23p19 antibodies are highly effective in reducing the Psoriasis Area Severity Index (PASI) score, skin lesions can reoccur during the treatment and after discontinued therapy, highlighting a gap in the understanding of the immune dysregulation occurring during psoriasis [4].

T helper 17 (Th17) cells are known to be the main IL-17 producers and involved in the pathogenesis of psoriasis in response to self-antigens such as LL37, an antimicrobial peptide (AMP) overproduced in psoriatic skin [5]. In addition, cytotoxic CD8^+^ T (Tc) cells can also express IL-17A (Tc17) upon recognition of the self-antigen ADAMTSL5 via HLA-C*06:02 which is the strongest psoriasis susceptibility allele in psoriasis patients revealing their pivotal pathogenic role in psoriasis [6, 7]. Before the identification of IL-17, disease severity was correlated with elevated levels of IFN-γ in serum and skin samples. Thus, the IFN-γ signaling pathway was considered the primary driving disease mechanism in psoriasis [8]. Successful therapies dampening IL-23/IL-17 axis re-defined the central role of IFN-γ to the early initiators of the psoriatic pathogenic cascade. IFN-γ blockade with fontolizumab downregulated DC-derived products, including IL-23p19, IL12/23p40, and iNOS confirming the role of IFN-γ in the early pathogenic steps [9]. IFN-γ is produced by auto-reactive Th and Tc cells in response to LL37 [5]. Furthermore, neutralizing IFN-γ in Tc cells blocked keratinocyte hyperproliferation in the psoriasis-like inflammation suggesting the unique pathogenic role of IFN-γ-producing Tc cells (Tc1) [8, 10]. The data from the publications mentioned above indicate the complexity of psoriasis development and the distinct pathogenic roles of multiple types of effector T cells, including IL-17A- and IFN-γ-producing Th and Tc cells.

Forkhead box protein 3 (Foxp3)^+^ regulatory T cells (Tregs) play a fundamental role in maintaining immune homeostasis and self-tolerance, and their instability and dysfunction result in excessive and uncontrolled immune responses leading to autoimmune diseases [11, 12]. Dysfunctional Tregs have reduced suppressive activity and can upregulate IL-17 expression, as identified in patients with psoriasis [13]. Consequently, reduced suppression of effector T cells might lead to an imbalance of Th17 and Tregs, associated with disease severity. As a result, the immune balance mediated by Tregs has been evaluated in potential psoriasis therapies. For instance, guselkumab (IL-23p19 blocker) treatment increased Tregs/CD8^+^ memory T cells (T_RM_) ratio, reaching superior long-term efficacy for treating moderate-to-severe psoriasis [14, 15]. Meanwhile, improvement of the immunomodulation mediated by Tregs has been intensively studied [16–21]. In line with observations from these studies, the beneficial effects of blocking pathogenic Th and Tc cells and restoring Tregs provide promising therapeutic strategies for psoriasis [16–21].

Individual T cell subsets utilize specific metabolic programs during differentiation and activation to generate rapid responses and fulfill their increasing demand for energy and biomass [22, 23]. These metabolic pathways are crucial for determining the immune cell state and fate decision providing the potential targets to modulate the balance between effector T cells and Tregs [22–24]. We have previously demonstrated that *de novo* fatty acid synthesis (FAS) is required for the differentiation of Th17 but not for the development of Tregs [24]. The Treg/Th17 balance can be shifted toward immune suppression by inhibiting the rate-limiting enzyme of FAS, acetyl-CoA carboxylase 1 (ACC1) [24, 25]. We tested here the therapeutic potential of targeting ACC1-mediated FAS in T cells in psoriasis. Emerging studies have revealed the use of context- and disease-specific metabolic profiles in subpopulations of Tregs for tissue adaptation [26–28] and indicate apparent differences in the metabolic requirements depending on the microenvironment and the activated signaling pathways, for instance, FAS is required for the maturation of intratumoral Tregs [26]. However, the role of FAS in Treg-mediated regulation of skin homeostasis and its potential effect on the functionality of Tregs during psoriasis have yet to be investigated.

In our current study, we utilized the imiquimod (IMQ)-induced mouse model of psoriasis (IMQ model) to analyze the role of FAS in T cells during psoriasis through the T cell-specific genetic ablation of ACC1. Our findings demonstrate that targeting FAS in T cells, ameliorates skin inflammation in the IMQ model by limiting IFN-γ-producing Th1/Tc1 and IL-17A-producing Th17/Tc17/dysfunctional Treg populations in the skin and frequencies of effector Tregs in the skin-draining LNs. Thus, inhibition of ACC1 in T cells could be a promising therapeutic approach for psoriasis treatment.

## Materials and methods

### Mice

Animal experiments were performed with C57BL/6J WT mice or the TACC1 mouse line which was previously generated by CD4^Cre/+^ mice crossed to ACC1^lox/lox^ mice and maintained on a C57BL/6J genetic background [24]. Their littermate CD4^Cre/wt^ACCl^fl/fl^ mice were used as WT controls. The mice were bred and housed in the animal facility of the University Medical Center of the Johannes Gutenberg-University of Mainz under specified pathogen-free conditions. All animal experiments were performed in compliance with the relevant guidelines and regulations for animal welfare by the federal state of Rhineland-Palatinate, Germany. Experiments were done with approval from the Landesuntersuchungsamt Rheinland-Pfalz (individual animal experimentation application no. G19-1-060), and efforts were made to minimize potential suffering of the mice.

### IMQ-induced psoriasis mouse model

Mice were shaved and depilated with hair removal cream (Veet^®^, Reckitt Benckiser Group, Slough, England) on the back skin 2 days before the treatment and then daily treated with 50 mg Aldara (containing 5% IMQ, purchased from Meda [Solna, Sweden]) or sham cream (without IMQ) on the back skin and 5 mg Aldara or sham cream per ear for both ears for six consecutive days as previously published [29]. For the back skin, skin thickness and disease severity were assessed daily with a scoring system for scaling and erythema in line with the human PASI. Erythema and scaling were scored from 0 to 4, with 0 indicating no severity and 4 indicating high disease severity. A cumulative score was generated from combined evaluation of parameters including erythema, scaling and thickness of the skin. Thickness was scored based on the increase in the back skin thickness of day 0 (1 for 20%-39%, 2 for 40-59%, 3 for 60%-79% and 4 for more than or equal to 80%) as previously published [29].

### Skin digestion protocol

Ears were separated into ventral and dorsal halves and cut into small pieces, followed by enzymatic digestion for 90 min at 37 °C with 4 mg/ml (1.2 U/ml) collagenase D (Sigma-Aldrich, St. Louis, MO), and 50 U/ml DNase I (Applichem, Darmstadt, Germany) in a gentleMACS™ Dissociator (Miltenyi Biotech).

### Flow cytometry

Single cell suspension was incubated with in-house Fc-receptor blocking reagent before staining of surface antigens. Dead cells were excluded by LIVE/DEAD Fixable Aqua Dead Cell Stain Kit (Invitrogen, Carlsbad, CA). For analysis of surface markers, cells were stained in PBS containing 0.25% BSA (Roche, Mannheim, Germany) and 0.02% NaN_3_ (Carl Roth GmbH+Co.KG, Karlsruhe, Germany). For the labelling of murine surface antigens, the following fluorescence-conjugated monoclonal antibodies were used: CD3e (145-2C11; eBiosciences, San Diego, CA), γδTCR (GL-3; eBiosciences, San Diego, CA), CD8a (Ly2; eBiosciences, San Diego, CA), CD45 (30-F11; BioLegend, San Diego, CA), CD25 (PC61.5; Thermo Fisher Scientific, Waltham, MA), CD4 (GK1.5; eBiosciences, San Diego, CA), CD62L (MEL-14; eBiosciences, San Diego, CA), CD44 (IM7; Thermo Fisher Scientific, Waltham, MA), CTLA-4 (UC10-4B9; eBiosciences, San Diego, CA), and TCR beta (H57-597; eBiosciences, San Diego, CA). For intracellular staining of cytokines and transcription factors, cells were stained with Foxp3 (FJK-16s; eBiosciences, San Diego, CA), IL-17A (eBio17B7; eBiosciences, San Diego, CA), IFN-γ (XMG1.2; eBiosciences, San Diego, CA), IL-10 (JES5-16E3; BD Biosciences, San José, CA, USA), KLRG1 (2F1/KLRG1; BioLegend, San Diego, CA) and GATA3 (TWAJ; eBiosciences, San Diego, CA) using the Foxp3/Transcription Factor Fixation/Permeabilization Kit (eBiosciences, San Diego, CA) according to the manufacturer’s instructions. As indicated in the respective experiments, cells were stimulated *in vitro* in the presence of phorbol-12-myristate-13-acetate (PMA) (0.1 μg/mL; Sigma-Aldrich, St. Louis, MO) and Ionomycin (1 μg/mL; Sigma-Aldrich, St. Louis, MO) for 2 h followed by incubation for 2 h with Brefeldin A (5 μg/mL; eBiosciences, San Diego, CA) before staining. Cells were acquired on Cytoflex S (Beckman Coulter) or Cytek Northern Lights (Cytek Biosciences, Fremont, CA), and data were analyzed with FlowJo software (Tree Star).

### Histology

For hematoxylin and eosin staining, tissue samples were fixed in 4% formaldehyde (Carl Roth GmbH+Co.KG, Karlsruhe, Germany) and embedded in paraffin. Images were taken using the microscope IX81 (Olympus, Shinjuku, Japan). The immune cell infiltration and histopathological scores were evaluated by a dermatopathologist. For immunohistochemistry staining of K17, paraffin fixed sections were stained with the following antibodies: primary antibodies: anti-Cytokeratin 17 (clone: EP1623; Abcam, Cambridge, UK) and secondary antibody goat-anti Rabbit (polyclonal, Sigma-Aldrich, St. Louis, MO). Nuclei were counterstained with Antifade Mounting Medium with DAPI (Novus Biologicals, Littleton, CO). The intensity of K17 was analyze by the software ZEN lite (Zeiss).

### Statistical analysis

Data analysis was performed with GraphPad Prism Software 9. *P values* were calculated using Student's t-test, one-way ANOVA, or two-way ANOVA as indicated. *P values* were considered significant as *p* < 0.05. Data are represented as means with standard deviations (SD).

## Results

### Genetic ablation of ACC1 in αβ T cells attenuates skin inflammation in the IMQ model

**Fig. 1:**
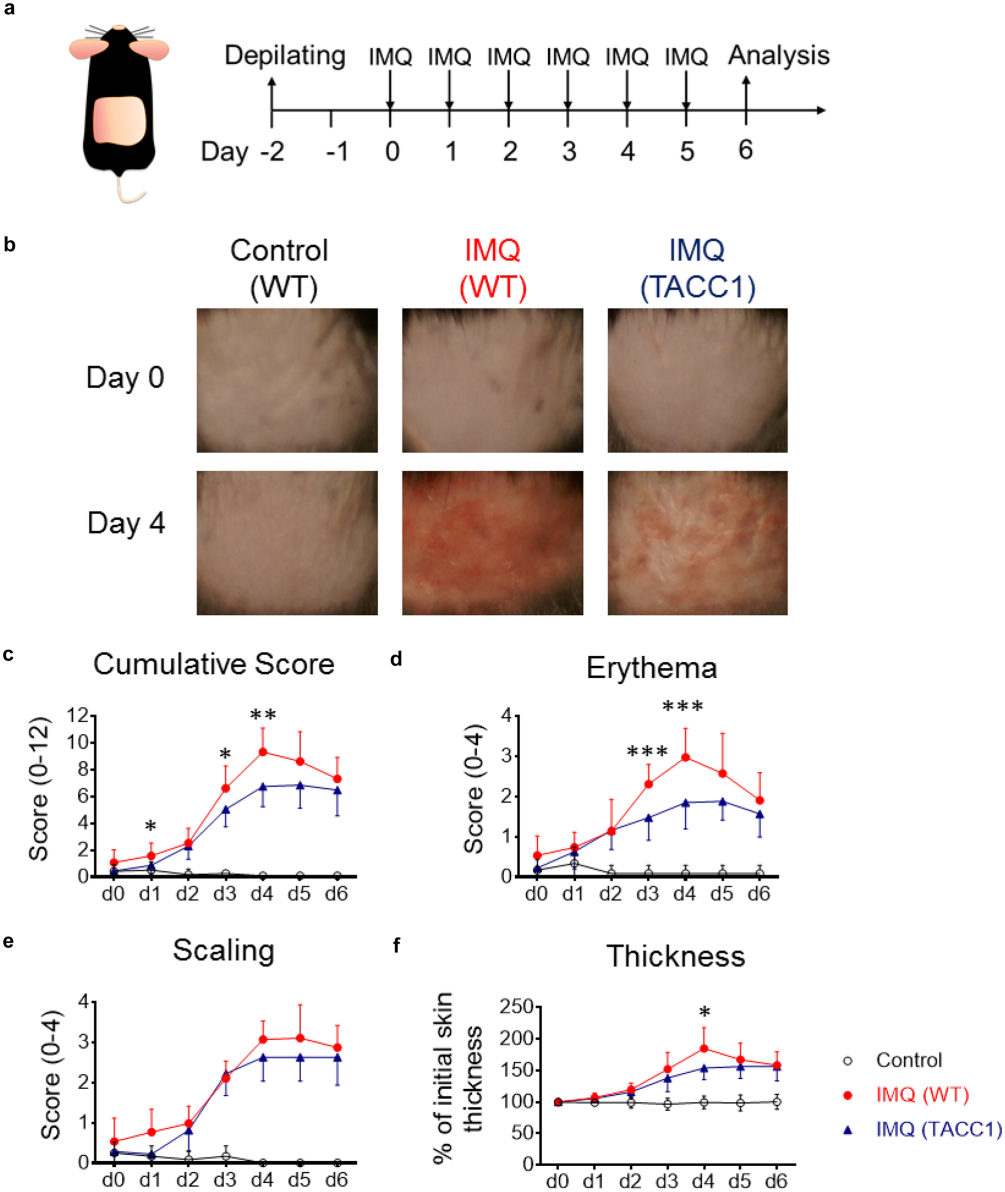
Genetic ablation of ACC1 in αβ T cells attenuates IMQ-induced skin inflammation (**a**) Schematic model of the experimental design for psoriasis induction. (**b**) Representative photos of the treated back skin area. (**c**) Cumulative scores of erythema (**d**), scaling (**e**), and thickness (**f**) were measured during the treatment. Pooled data from four independent experiments including groups of Control = WT mice treated with control cream (n=6), IMQ (WT) = WT mice treated with IMQ (n=15), and IMQ (TACC1) = T cell-specific ACC1–deficient mice (TACC1 mice) mice treated with IMQ (n=16). Error bars represent SD. *P-values* were determined by two-way ANOVA comparing IMQ (WT) and IMQ (TACC1) (* *p*<0.05, ** *p*<0.01, *** *p*<0.001)

**Fig. 2:**
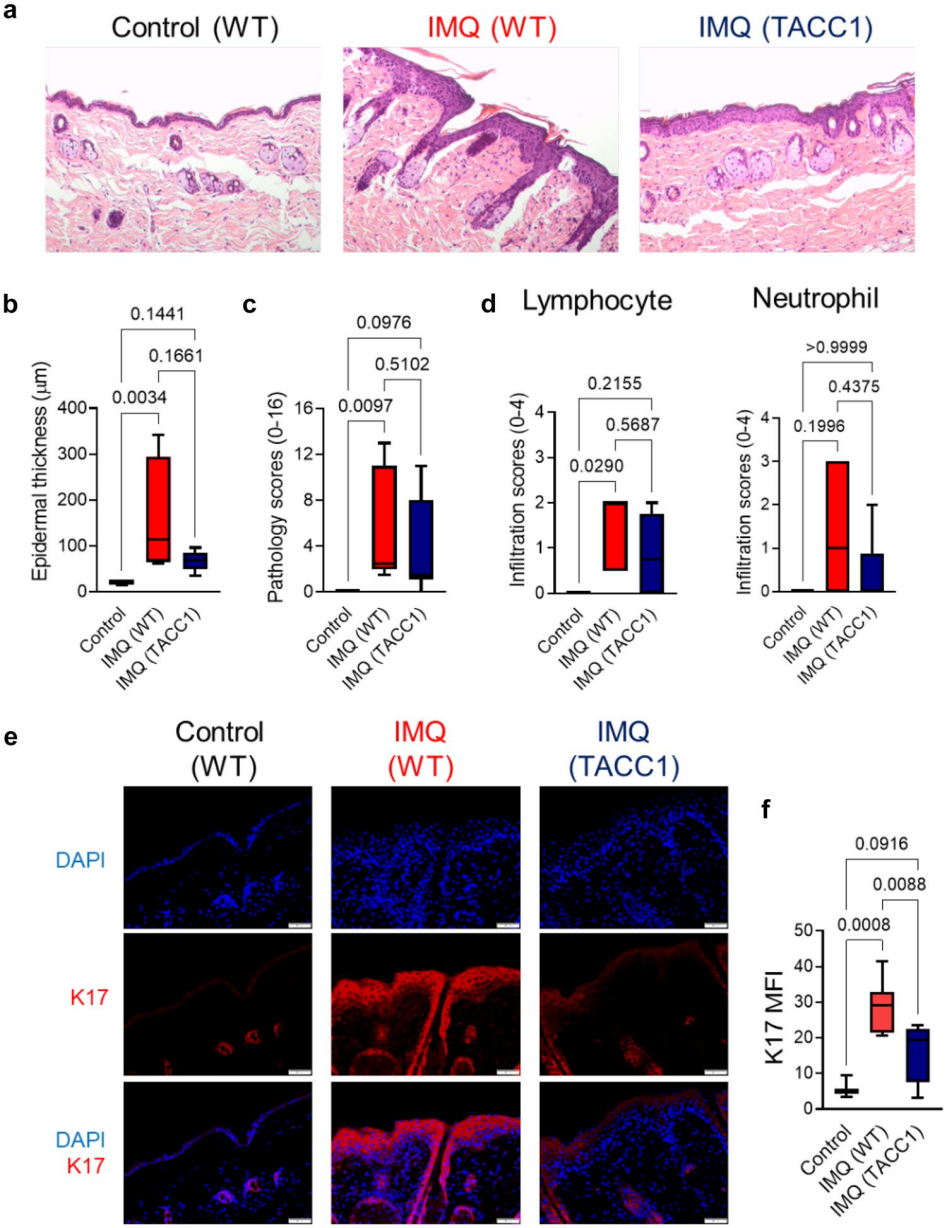
Genetic ablation of ACC1 in αβ T cells mildly attenuates keratinocyte hyperproliferation in the IMQ model. (**a**) Representative histological sections were collected from the treated back skin area on day 6, as indicated in Fig. 1a. Epidermal thickness (**b**), histopathological scores (**c**), and immune cell infiltration (**d**) were determined in histological back skin sections, as shown in (**a**). (**e**) Immuno-staining with anti-keratin 17 (K17) antibody of the back skin from TACC1 or WT littermate control mice. (**f**) Mean fluorescence intensity (MFI) of K17 as shown in (**e**). Control = WT mice treated with control cream (n=3), IMQ (WT) = WT mice treated with IMQ (n=7), and TACC1 mice treated with IMQ (n=11). Error bars represent SD. *P-values* were obtained by one-way ANOVA

To investigate whether *de novo* FAS in T cells is required to develop psoriatic skin inflammation, we used our previously generated T cell-specific ACC1–deficient mice (TACC1 mice) [24, 25] in parallel to WT mice in the well-established IMQ model (Fig. 1a) [29–31]. Genetic ablation of ACC1 driven by CD4-Cre expression in mice carrying a loxP-flanked biotin carboxyl carrier protein domain in the *Acaca* gene (ACC1^fl/fl^) resulted in defective ACC1 mediated-FAS in αβ T cells (including both CD4^+^ and CD8^+^ T cells) [24]. Daily topical IMQ treatment induced skin inflammation accompanied by increased immune cell infiltration and hyperproliferation of keratinocytes (Figs. 1b-f and 2a-d). After two days of IMQ treatment, both WT and TACC1 mice developed erythema and scaling (Fig. 1d, e). However, by day 3, IMQ-treated TACC1 mice showed significantly less erythema than IMQ-treated WT mice (Fig. 1d). In WT mice, IMQ-associated skin inflammation reached the peak by day 4, demonstrated by cumulative scores of erythema, scaling, and skin thickness (Fig. 1c). TACC1 mice showed significantly lower scores at the peak of inflammation due to considerably reduced erythema and skin thickness, although no significant changes in scaling were observed compared to the IMQ-treated WT mice (Fig. 1d-f).

We further determined the degree of keratinocyte proliferation as depicted by epidermal thickness, infiltration of lymphocytes and neutrophils, histopathologic scores of psoriasis (acanthosis, parakeratosis, hypogranulosis, and Munro's microabscess), and the expression of Keratin 17 (K17), a marker of keratinocyte hyperproliferation in psoriasis (Fig 2a-f) [3]. Given the time course for the induction of IL-17-mediated keratinocyte hyperproliferation, we analyzed skin sections collected from mice on day 6 of the IMQ model [3, 29]. At this time point, IMQ significantly induced epidermal thickening and increased histopathologic scores of psoriasis in WT mice compared to control cream-treated mice (Fig. 2b, c). In line with the reduced clinical scores (Fig. 1), IMQ-treated TACC1 mice showed slightly reduced epidermal thickening and histopathologic scores compared to IMQ-treated WT mice (Fig. 2a-c). At day 6, however, only modest lymphocyte and neutrophil infiltration levels were observed in the IMQ-treated groups (Fig. 2d). The K17 level was remarkably increased in IMQ-treated WT skin versus control cream-treated skin, while TACC1 mice showed significantly reduced K17 expression (Fig. 2e, f). Taken together, genetic ablation of ACC1 in αβ T cells attenuates psoriatic disease and keratinocyte hyperproliferation in the IMQ model.

### ACC1 deficiency restrains IL-17A and IFN-γ producing cells induced in the IMQ model

**Fig. 3:**
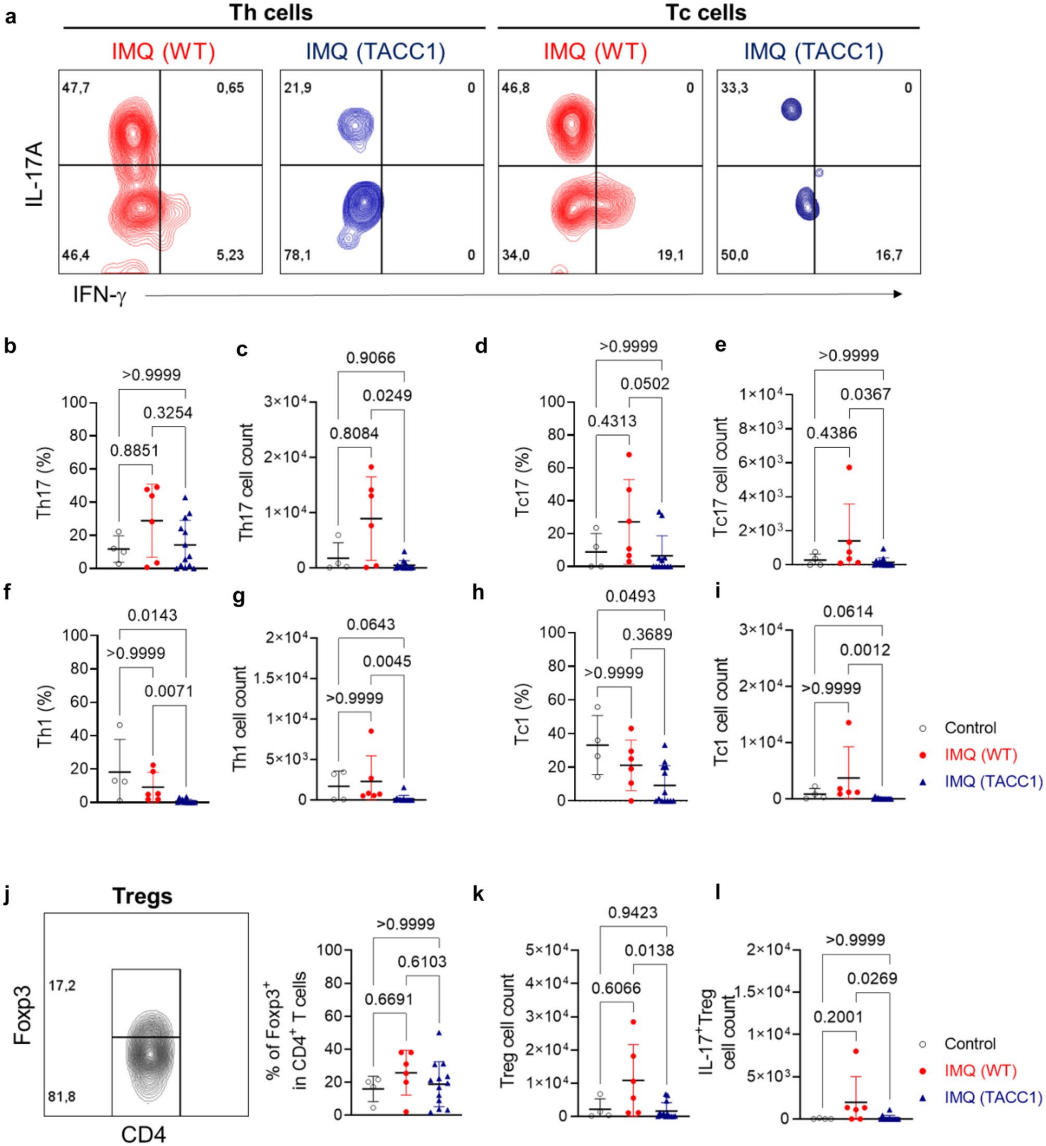
Genetic ablation of ACC1 in αβ T cells limits Tc cell infiltration in local skin area during IMQ-mediated psoriatic induction. T cells were isolated from back skin on day 4 of the IMQ model, as indicated in Fig. 1a. (**a**) Representative gating strategy of flow cytometry analysis for percentages of IL-17A and IFN-γ in Th (CD3^+^αβTCR^+^CD4^+^Foxp3^-^) and Tc (CD3^+^αβTCR^+^CD8^+^) cells. Percentages of IL-17A^+^ in Th cells (**b**) and normalized quantification of Th17 cells (**c**) isolated from treated skin. Percentages of IL-17A^+^ in Tc cells (**d**) and normalized quantification of Tc17 cells (**e**) isolated from treated skin. Percentages of IFN-γ^+^ in Th cells (**f**) and normalized quantification of Th1 cells (**g**) isolated from treated skin. Percentages of IFN-γ^+^ in Tc cells (**h**) and normalized quantification of Tc1 cells (**i**) isolated from treated skin. (**j-l**) Representative gating strategy of flow cytometry analysis for Foxp3 percentages and cell counts of Tregs and IL-17A^+^Tregs (CD3^+^αβTCR^+^CD4^+^Foxp3^+^IL-17A^+^). Cell counts were normalized to tissue weight (50 mg of each sample). Pooled data from three independent experiments including groups of Control = WT mice treated with control cream, IMQ (WT) = WT mice treated with IMQ, and TACC1 mice treated with IMQ. Error bars represent SD. *P-values* were obtained by one-way ANOVA with Kruskal-Wallis-Test

ACC1 deficiency in αβ T cells mainly affected the pathological outcome at the peak of the disease. Therefore, we focused on an in-depth analysis of αβ T cells at the peak of inflammation on day 4 of the IMQ model. To gain an overview of the infiltrating αβ T cells in IMQ-treated back skin, we analyzed different functional subsets of αβ T cells, including IL-17-producing Th17 (Fig. 3a-c) and Tc17 (Fig. 3d, e), IFN-γ-producing Th1 (Fig. 3f, g) and Tc1 (Fig. 3h, i), and Tregs (Fig. 3j-l). IMQ-treated WT mice displayed mild tendencies of increased infiltrating Th17 and Tc17 cells and similar Th1 and Tc1 cells compared to control cream-treated mice (Fig. 3b-i). However, compared to IMQ-treated WT mice, Th17 (Fig. 3c), Tc17 (Fig. 3e), Th1 (Fig. 3g), and Tc1 (Fig. 3i) cells were significantly reduced in the skin of IMQ-treated TACC1 mice.

Unlike the IMQ-treated back skin, IMQ treatment only induced small proportions of Th and Tc cells to produce IL-17A in the skin-draining LNs (Supplementary Fig. S1). The frequencies of IL-17A-producing Th17 and Tc17 cells were comparable between IMQ-treated WT and TACC1 mice (Supplementary Fig. S1b, c). Nevertheless, TACC1 mice showed tendencies of lower Th17 and Tc17 cell numbers in the skin-draining LNs (Supplementary Fig. S1d, e). Similarly, only a small proportion of Th cells produced IFN-γ in the skin-draining LNs (Supplementary Fig. S1f, g). IMQ-treated WT mice displayed slightly increased frequencies of IFN-γ-producing Th1 cells compared to all the other groups (Supplementary Fig. S1f). In WT mice, most Tc cells produced IFN-γ and less IL-17A in skin-draining LNs (Supplementary Fig. S1g). TACC1 mice showed significantly reduced frequencies of IFN-γ-producing Tc1 cells compared to IMQ-treated WT mice (Supplementary Fig. S1g). Furthermore, the absolute cell numbers of Th1 (Supplementary Fig. S1h) and Tc1 cells were significantly reduced in the skin-draining LNs of TACC1 mice compared to IMQ-treated WT mice (Supplementary Fig. S2i). Surprisingly, the frequencies of Tregs in the IMQ-treated skin of TACC1 compared to WT mice were comparable (Fig. 3j). Of note, IMQ-treated WT mice had more Tregs in the skin compared to TACC1 mice (Fig. 3k). However, there were significantly increased Tregs co-expressing IL-17A in IMQ-treated WT mice but not in IMQ-treated TACC1 mice (Fig. 3l). Therefore, these findings suggest that genetic ablation of ACC1 in T cells dampens IMQ-mediated skin inflammation mainly by limiting IL-17A production from Th17, Tc17, and IL-17A^+^Treg and IFN-γ production from Th1 and Tc1 populations rather than promoting suppressive Tregs in the skin on day 4 of the IMQ model.

### ACC1 deletion in T cells increases effector tregs under steady-state and psoriatic conditions

**Fig. 4:**
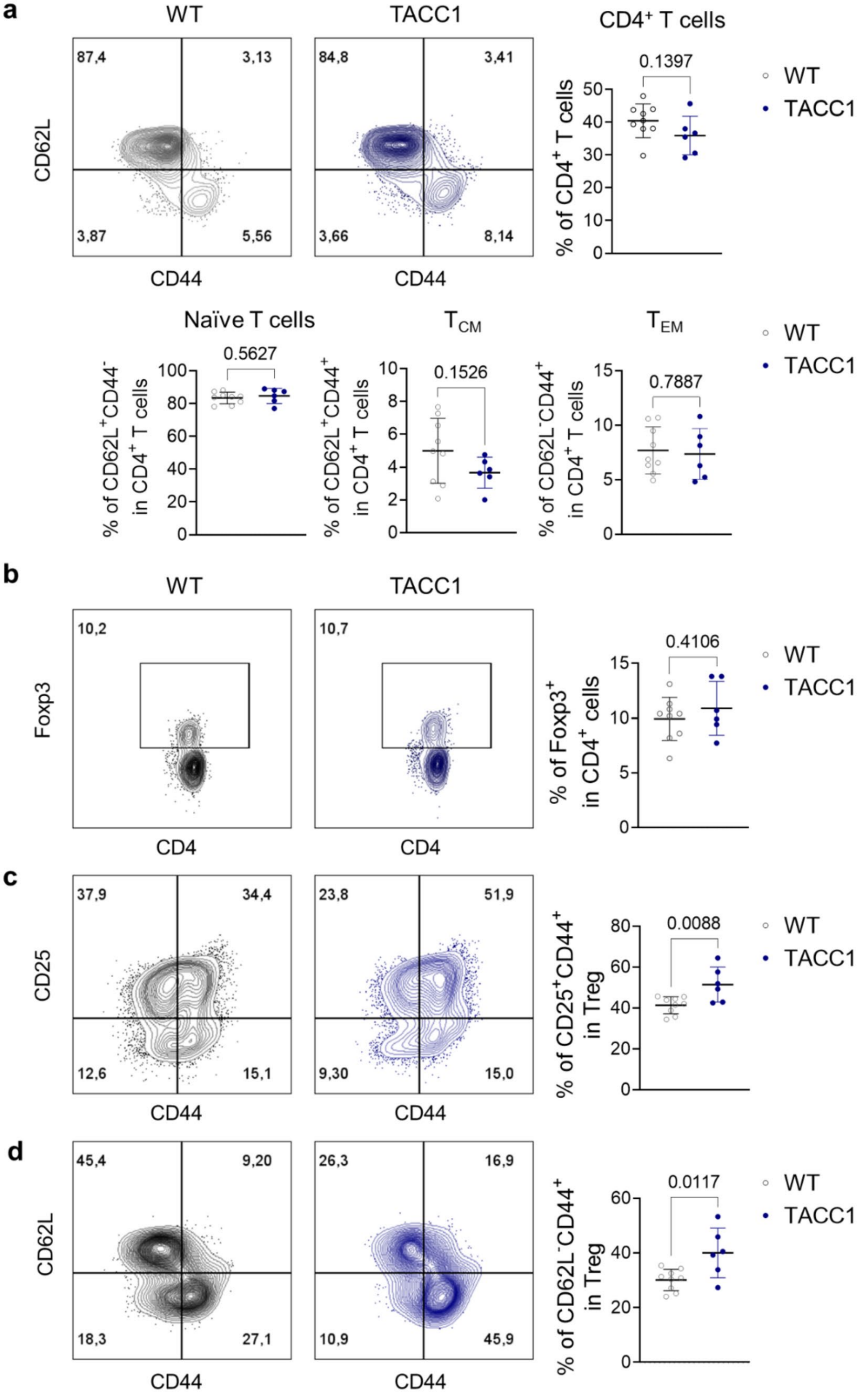
Genetic ablation of ACC1 in αβ T cells increases effector Tregs in skin-draining LNs during homeostasis. T cells were isolated from inguinal LNs under steady-state conditions. (**a**) Representative flow cytometry analysis and frequencies of CD62L^+^CD44^-^ Naïve T cells, CD62L^+^CD44^+^ T_CM_, and CD62L^-^CD44^+^ T_EM_ in the skin-draining LNs. (**b**) Representative flow cytometry analysis and percentages of Tregs in LNs. (**c**) Representative flow cytometry analysis and CD44 and CD25 expression frequencies in Tregs isolated from LNs. (**d**) Representative flow cytometry analysis and CD62L and CD44 expression percentages in Tregs from LNs. Data was collected from WT (n=9) and TACC1 mice (n=6). Error bars represent SD. *P-values* were obtained by t-test

We have previously shown that TACC1 mice display slightly reduced peripheral T cells with normal frequencies of Th17 and Tregs in lymphoid organs during homeostasis [24]. To understand whether genetic ablation of ACC1 in T cells provides an initial advantage in modulating unwanted psoriatic inflammation, we further evaluated the phenotypes of Tregs. We confirmed a tendency of decreased CD4^+^ T cells in the skin-draining LNs (Fig. 4a) as published [24]. However, we did not observe any changes in the composition of naïve T cells (CD62L^+^CD44^-^), central memory T cells (CD62L^+^CD44^+^; T_CM_), and effector memory T cells (CD62L^-^CD44^+^; T_EM_) in the skin-draining LNs during homeostasis (Fig. 4a). Next, we evaluated Tregs during homeostasis. In the absence of inflammation, the percentages of Tregs in the LN were comparable between WT and TACC1 mice (Fig. 4b). Notably, when we investigated effector subsets of Tregs within the skin-draining LNs, TACC1 mice had a significantly higher proportion of activated CD44^+^CD25^+^ Tregs and effector CD62L^-^CD44^+^ Tregs (Fig. 4c, d) which can support tissue protection in the absence of inflammation [32, 33]. Tregs residing in the LNs downregulate CD62L to reduce adhesion to high endothelial venules in LNs and upregulate CD44, a receptor to extracellular matrix ligands, to facilitate their trafficking to nonlymphoid tissues [32, 33]. Those effector phenotypes could grant them the capacity to respond rapidly to tissue damage and immune-mediated inflammation such as psoriasis induction as we observed in the IMQ-treated TACC1 mice.

**Fig. 5:**
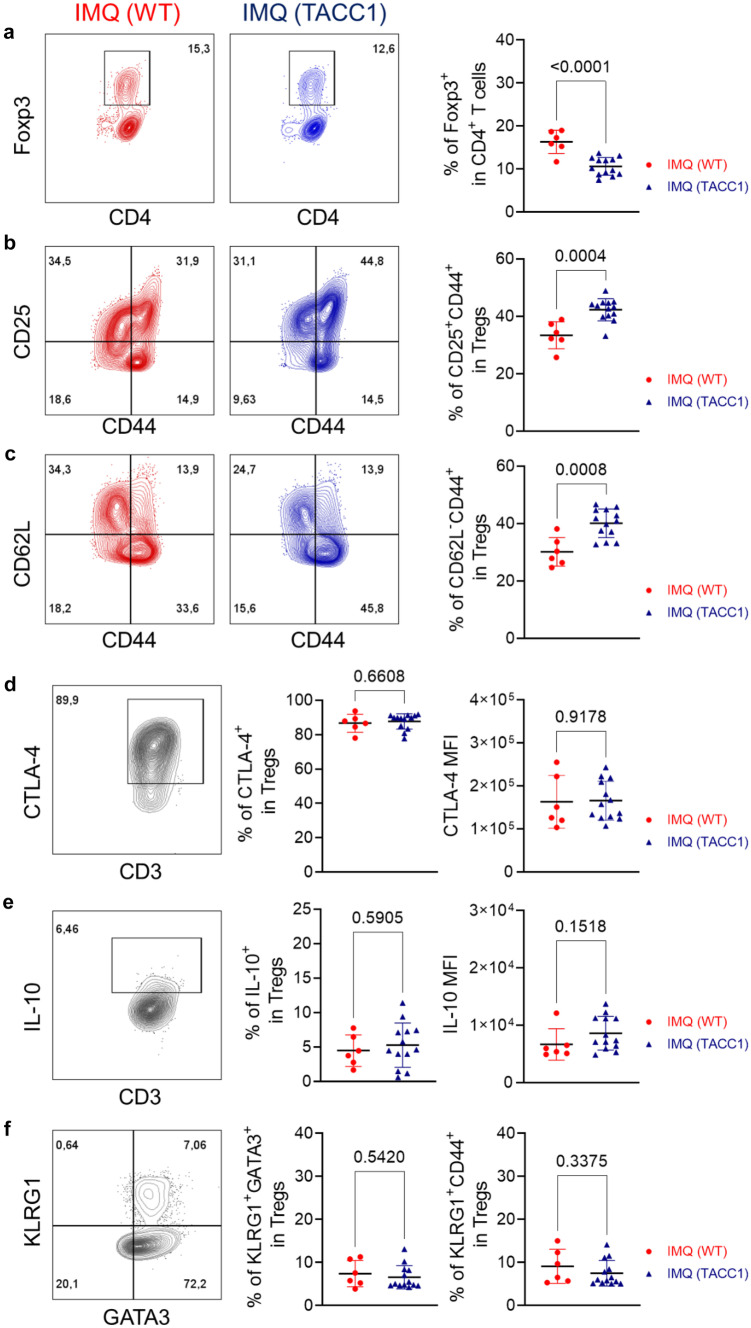
Genetic ablation of ACC1 in αβ T cells increases effector Tregs in the skin-draining LNs during IMQ-mediated psoriasis. T cells were isolated from the skin-draining LNs on day 4 of the IMQ model, as indicated in Fig. 1a. (**a**) Representative flow cytometry analysis and percentages of Tregs. (**b**) Representative flow cytometry analysis and CD44 and CD25 expression percentages in Tregs. (**c**) Representative flow cytometry analysis and CD62L and CD44 expression percentages in Tregs. (**d**) Representative flow cytometry analysis of percentages and MFI of CTLA-4 in Tregs. (**e**) Representative flow cytometry analysis of percentages and MFI of IL-10 in Tregs. (**f**) Representative flow cytometry analysis and percentages of KLRG1, GATA3, and CD44 expression in Tregs. Data was collected from WT (n=6) and TACC1 mice (n=13). Error bars represent SD. *P-values* were obtained by t-test

We further assessed whether TACC1 mice maintained high frequencies of activated CD44^+^CD25^+^ Tregs and effector CD62L^-^CD44^+^ Tregs compared to the WT mice during the IMQ application. Of note, after 4 days of psoriasis induction, IMQ-treated WT mice increased the frequency of Tregs in the skin-draining LNs compared to steady-state conditions (Figs. 4b and 5a). In contrast, the frequency of Tregs in IMQ-treated TACC1 mice was similar to the homeostatic condition (Figs. 4b and 5a), probably due to the attenuated inflammation induced by IMQ during the early phase in TACC1 mice (Figs. 4b and 5a). Nevertheless, among these Tregs, TACC1 mice maintained significantly higher proportions of activated CD44^+^CD25^+^ Tregs and effector CD62L^-^CD44^+^ Tregs (Fig. 5b, c).

We further checked the suppressive functions of these Tregs and thus assessed frequencies and expression levels of CTLA-4 and IL-10 in the skin-draining LNs of IMQ-treated mice (Fig. 5d, e). However, ACC1-deficient Tregs showed comparable IL-10 and CTLA-4 expression levels to WT Tregs (Fig. 5d, e). Furthermore, tissue Tregs expand after tissue injury to promote tissue regeneration and repair. These tissue Treg generation required priming in the secondary lymphoid organs to express killer cell lectin-like receptor G1 (KLRG1) before reaching the final acquisition of tissue-specific signatures [34–36]. KLRG1 binds E-cadherin, a key component of epithelial intercellular junctions, specifically associated with Tregs expressing GATA3, a preferential transcription factor expressed by skin Tregs [37]. TACC1 mice showed comparable frequencies of GATA3^+^KLRG1^+^ and KLRG1^+^CD44^+^ tissue Treg precursors compared to IMQ-treated WT mice in the skin-draining LNs (Fig. 5f). Thus, genetic ablation of ACC1 in Tregs mainly promoted effector Tregs under steady state and psoriatic conditions in the skin-draining LNs.

**Fig. 6:**
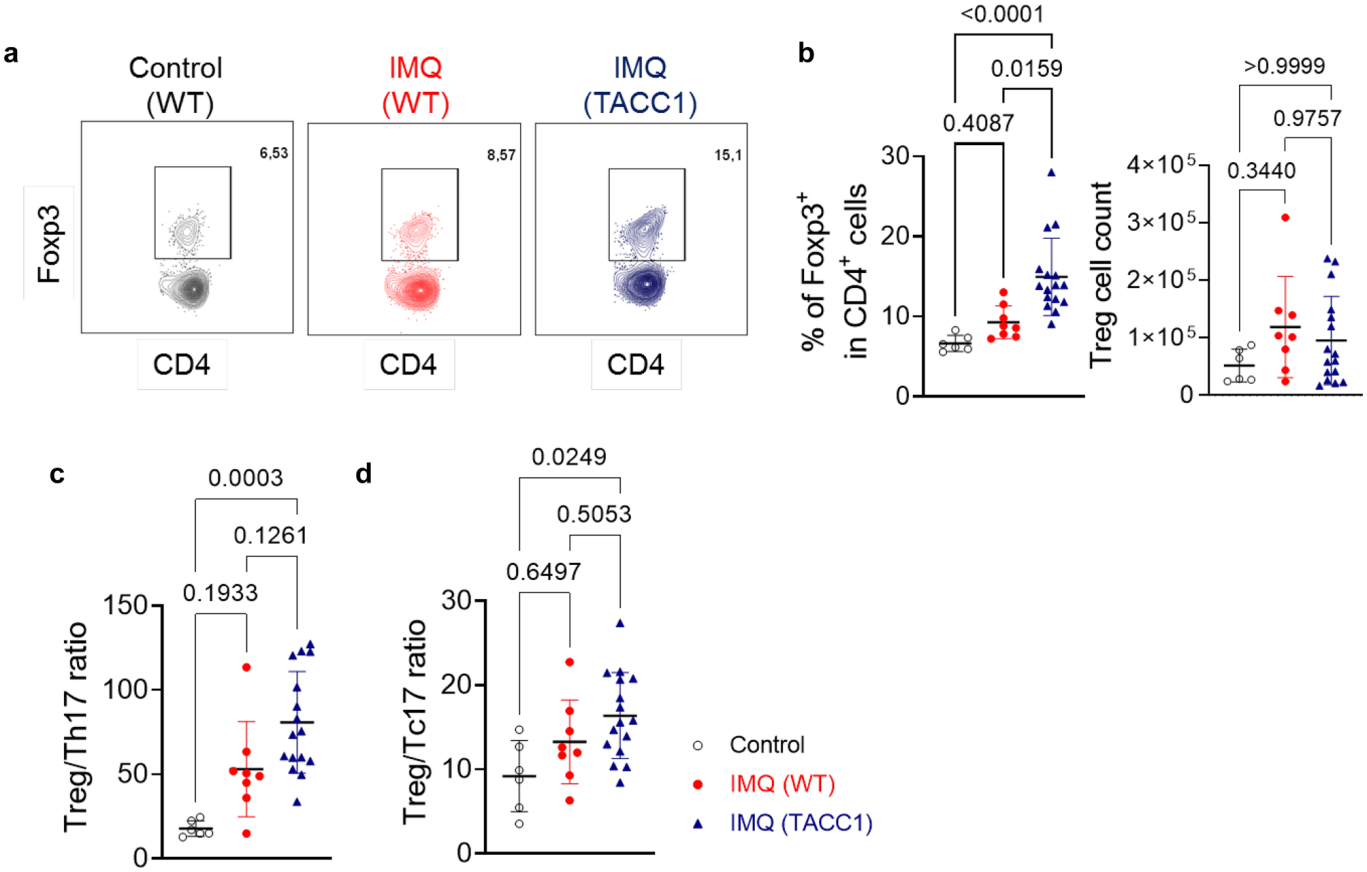
Percentages and cell counts of Tregs in the skin-draining LNs during IMQ-mediated inflammation. T cells were isolated from skin-draining LNs on day 6, as indicated in Fig. 1a, and stimulated with PMA/Ionomycin to determine cytokine expression. (**a** and **b**) Representative flow cytometry analysis of Treg (CD45^+^CD3^+^CD4^+^Foxp3^+^) within CD4^+^ T cells frequencies and Treg cell count from skin-draining LNs. Ratios of Tregs to Th17 (**c**) and Tc17 (**d**). Pooled data from three independent experiments. Error bars represent SD. *P-values* were obtained by one-way ANOVA with Kruskal-Wallis-Test

Notably, by day 6, IMQ-treated TACC1 mice displayed a significantly higher percentage of Tregs upon stimulation than WT mice (Fig. 6a, b). A Treg/Th17 imbalance is associated with higher psoriasis severity [13]. However, IMQ-treated WT mice did not show Treg/Th17 and Treg/Tc17 imbalances on day 6, probably due to a resolution of αβ T cell-mediated inflammation by day 6 (Fig. 6c, d). Nevertheless, IMQ-treated TACC1 mice showed tendencies of increased Treg/Th17 and Treg/Tc17 ratios compared to WT mice (Fig. 6c, d), suggesting ACC1 deficiency promotes Treg-mediated immune balance.

Interestingly, IMQ treatment resulted in a significant expansion of IL-17A-producing γδ T cells in the skin-draining LNs of both WT and TACC1 mice compared to the control cream-treated WT mice (Supplementary Fig. S2a, b). Thus, this IL-17A-producing T cell population might contribute to the low inflammation observed on day 6 in TACC1 mice (Fig. 1c-f).

In summary, we demonstrated that genetic ablation of ACC1-mediated FAS in T cells promotes effector Tregs under steady-state conditions. Upon psoriasis development, TACC1 mice maintained higher frequencies of effector Tregs and limited IFN-γ-producing Th1/Tc1 in skin-draining LNs. In IMQ-treated skin, ACC1 deficiency in αβ T cells restrains not only IL-17A-producing Th17/Tc17/dysfunctional Treg but also IFN-γ-producing Th1/Tc1 populations in the psoriatic skin lesions resulting in attenuated inflammation, thus offering a potential therapeutic target for clinical use.

## Discussion

In the current study, we demonstrate that T-cell-specific deletion of ACC1 limits IL-17A-producing Th17/Tc17/dysfunctional Treg and IFN-γ-producing Th1/Tc1 populations and, as a result, ameliorated psoriatic skin inflammation. Several studies have determined the pathogenic roles of Th17/Th1/Tc17/Tc1 in psoriasis. However, little is known about how to improve their regulation by Tregs [16–21]. We observed that effector Treg frequencies were remarkably increased in TACC1 mice under homeostasis and maintained during psoriasis development. Tregs have been shown to suppress the infiltration of Tc and Th cells in preclinical psoriatic mouse models, which might partially explain the reduced Tc and Th populations in skin lesions observed in IMQ-treated TACC1 mice [16, 17]. In particular, ACC1 deficiency in αβ T cells limited IL-17A and IFN-γ production by decreasing the absolute cell numbers of Th17/Th1/Tc17/Tc1 infiltrating in the skin. ACC1 also controlled dysfunctional IL-17A-producing Tregs in psoriatic lesions. Furthermore, ACC1 deletion reduced the frequencies of IFN-γ-producing Tc1 cells in skin-draining LNs. Taken together, targeting ACC1 in αβ T cells abolished the IL-17A and IFN-γ-producing αβ T cells and promoted effector Tregs to restrain psoriatic skin inflammation in the IMQ model.

IMQ rapidly triggers psoriasis development by activating TLR7 on dendritic cells to produce pro-inflammatory cytokines, including IL-23 and IL-1β, without directly providing specific antigens to be presented through MHCI and MHCII. In response, acute IL-17 production is produced from innate-like γδ T cells. However, IL-17A can induce keratinocytes to express K17, which is abnormally overexpressed in psoriasis. K17 contains peptides that trigger autoreactive T cells. This could lead to the development of Th1/Th17/Tc1/Tc17 cells [3]. The pro-inflammatory cytokines produced by these T cells act back on keratinocytes to promote their hyperproliferation [3]. Accordingly, keratinocytes and T cell cytokine production drive an autoimmune loop, resulting in the psoriasis phenotype [3]. Our results demonstrated that interfering with FAS in αβ T cells mainly affects the early phase of psoriasis induction, suggesting the early effect on blocking cytokine production from self-reactive αβ T cells (Fig. 1). Later on, expanded innate-like γδ T cells provide dominant IL-17A production contributing to the remaining inflammation observed in the IMQ-treated TACC1 mice (Figs. 1 and S2).

Under steady-state conditions, TACC1 mice have higher proportions of activated CD44^+^CD25^+^ Tregs and effector CD62L^-^CD44^+^ Tregs in skin-draining LNs that could respond rapidly to control inflammation in tissue (Fig. 4) [32, 33]. During psoriasis induction, TACC1 mice maintained significantly higher proportions of activated CD44^+^CD25^+^ Tregs and effector CD62L^-^CD44^+^ Tregs in skin-draining LNs (Fig. 5). They could contribute to suppressing auto-reactive effector T cells in the initial stage of psoriasis induction. In addition to activation status, these Tregs highly express CD25 to compete with conventional T cells for the uptake of IL-2, limiting the proliferation of the latter [38]. Furthermore, we demonstrated that these effector Tregs maintained in TACC1 mice displayed suppression functions normally by IL-10 and CTLA-4 (Fig. 5d, e). Thus, TACC1 mice may have an initial advantage in controlling inflammation due to this predisposed increase in functional effector Tregs in skin-draining LNs. After IMQ treatment, TACC1 mice also demonstrated a higher percentage of Foxp3^+^ Tregs in the skin-draining LNs than WT mice on day 6 (Fig. 6b). We speculated, based on our previous work [24], that an increased frequency of Tregs in TACC1 mice could be caused indirectly by the blockade of Th17 differentiation. Nevertheless, we did not observe an increased frequency of Tregs in the skin nor skin-draining LNs of IMQ-treated TACC1 mice by day 4. Therefore, our findings might suggest an alternative earlier role of cell-intrinsic FAS in effector Tregs. In addition, it is well documented that IMQ treatment induces acute inflammation in contrast to the chronic inflammation observed in psoriasis patients. Th cell levels are reduced on day 6 in IMQ-mediated skin inflammation from C57BL/6J mice, which may limit the effect of increased Treg frequency obtained in this study [39]. Therefore, to explore the clinical potential of ACC1 inhibition in inflammatory skin disease, in the future, we plan to investigate the use of topical inhibitors of ACC1 such as Soraphen A (SorA) [24] and its effects on human T cells isolated from psoriasis patients.

A possible mechanism to promote Tregs directly by ACC1 deletion is interfering with the intracellular level of acetyl-CoA. ACC1 catalyzes the carboxylation of acetyl-CoA to malonyl-CoA. Therefore, ACC1-deletion leads to the cytoplasmic accumulation of its substrate, acetyl-CoA [40], which, in turn, can be used by the cell for epigenetic regulation, such as histone acetylation. Histone acetylation catalyzed by CBP/p300 is indispensable for Treg development, and acetylation of the Foxp3 protein itself is required to prevent ubiquitin-mediated proteasomal degradation [41–43]. Quantifying intracellular levels of acetyl-CoA and acetylation of Foxp3 and other specific transcription regulators involved in Treg development and function could provide more insights into the underlying mechanism of ACC1 inhibition and its clinical application in autoimmune disease.

We have previously demonstrated that *de novo* FAS is required to differentiate Th1 and Th17 [24]. Others have demonstrated that ACC1-mediated FAS is essential for peripheral Tc cell homeostatic proliferation and antigen-specific response upon listeria infection [44]. We further uncovered the role of ACC1 in controlling the infiltration of Th1/Th17 and Tc1/Th17 in skin autoimmune disease, psoriasis. It is well appreciated that cell-intrinsic FAS is indispensable for the survival of T_RM_ cells, one of the populations of Tc cells in the skin under homeostatic conditions [45]. Yet, the complete role of cell-intrinsic FAS that is required for T_RM_ cell expansion during psoriatic inflammation remains to be confirmed by secondary challenge with IMQ. Interestingly, genetic ablation of ACC1 inhibited IL-17A-producing Tregs in the IMQ-treated skin lesions compared to IMQ-treated WT mice (Fig. 3l). Similarly, the ACC1 deficiency also restrained IL-17A-producing Th17/Tc17, potentially implying common mechanisms by which ACC1-mediated FAS favors the IL-17A production in Th17/Tc17 and dysfunctional Treg populations (Fig. 3c, e, and l) [46]. Due to the Cre line used here targeting αβ T cells, changes in γδ T cells will be considered as indirect regulations by αβ T cells, including Tregs [47]. TACC1 mice did not show changes to IL-17A-producing γδ T cells, which could be mainly responsible for the remaining scaling and inflammation. Further studies regarding the regulation of IL-17 production from γδ cells and the metabolic processes involved would be essential to understand how IL-17A production is controlled in γδ T cells. Furthermore, ACC1 is required to upregulate IL-22 expression levels in RORγt^+^ innate lymphoid cells (ILC3), which may also promote inflammation in psoriasis [48]. Accordingly, it could be beneficial to develop a broad pharmacological ACC1 inhibitor to target both conventional T cells and ILCs for psoriasis therapy. The topical use of SorA as a potential therapeutic agent [24] is subject to ongoing experiments. Taken together, our study demonstrated that ACC1 deletion in T cells has strong anti-inflammatory effects in IMQ-induced psoriasis, suggesting that pharmacological inhibition of FAS could provide novel therapeutic avenues for psoriasis therapy.

## Supplementary Information

Below is the link to the electronic supplementary material.

Supplementary file1 (DOCX 311 KB)

## Data Availability

The datasets generated during and/or analyzed during the current study are available from the corresponding author upon reasonable request.
