# Supplementary material for: Targeting ACC1 in T cells ameliorates psoriatic skin inflammation
Source: J Mol Med (Berl). 2023 Aug 18;101(9):1153–66. doi: 10.1007/s00109-023-02349-w (PMC10482807; doi:10.1007/s00109-023-02349-w)
Supplement: Supplementary file 1 — Supplementary file1 (DOCX 311 KB) [file 109_2023_2349_MOESM1_ESM.docx]

**Supplementary Figures**


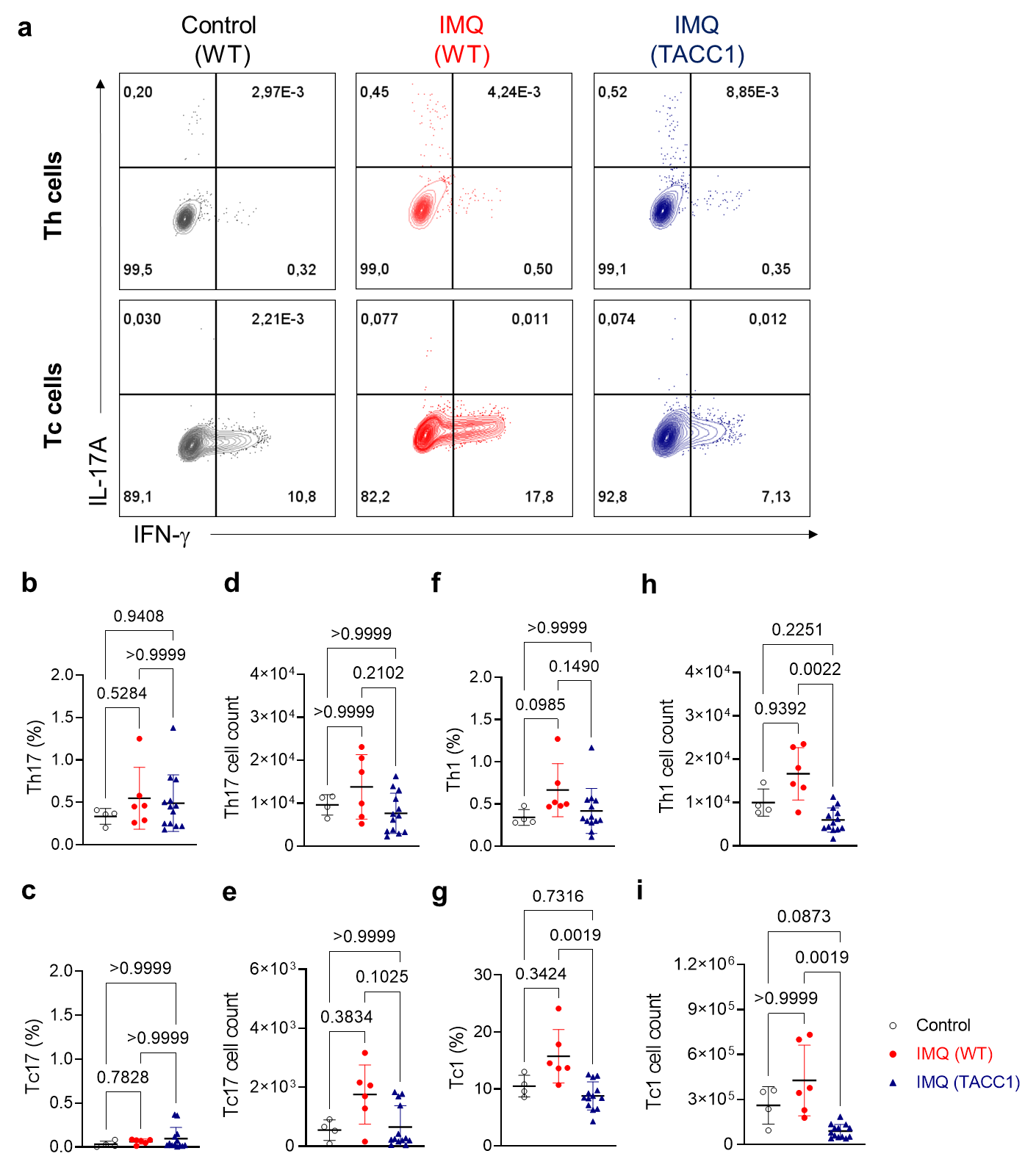


**Supplementary Fig. 1 Percentages and cell counts of Th17, Th1, Tc17, and Tc17 cells in the skin-draining LNs on day 4 of the IMQ model.** T cells were isolated from skin-draining LNs on day 4, as indicated in **Fig. 1a,** and stimulated with PMA/Ionomycin to determine cytokine expression. **(a)** Representative flow cytometry analysis of IL-17A and IFN-γ expression in Th (CD3^+^αβTCR^+^CD4^+^Foxp3^-^) and Tc (CD3^+^αβTCR^+^CD8^+^) cells. Percentages of IL-17A^+^ in Th **(b)** and Tc **(c)** cells. Cell counts of IL-17A^+^ Th17 **(d)** and Tc17 **(e)** cells**.**  Percentages of IFN-γ in Th **(f)** and Tc **(g)** cells. Cell counts of IFN-γ^+^ Th1 **(h)** and Tc1 **(i)** cells**.** Pooled data from three independent experiments. Error bars represent SD. *P-values* were obtained by one-way ANOVA with Kruskal-Wallis-Test.


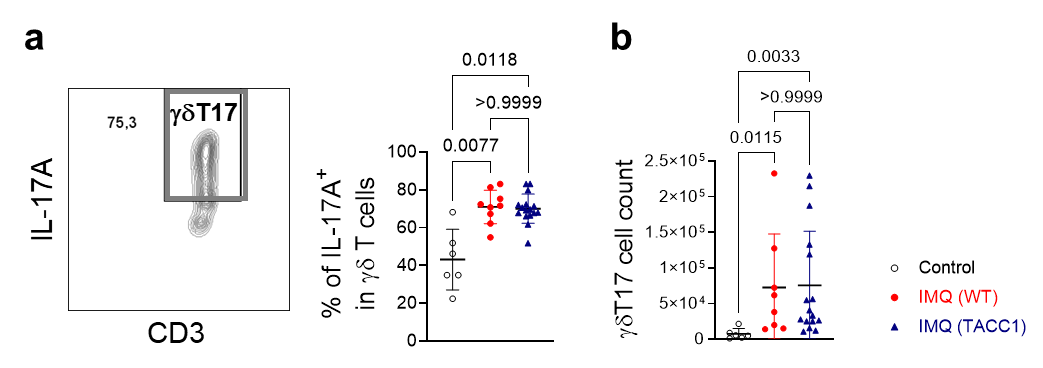


**Supplementary Fig. 2 Percentages and cell counts of IL-17A^+^γδ T cells in the skin-draining LNs during IMQ-mediated inflammation.** T cells were isolated from skin-draining LNs on day 6, as indicated in **Fig. 1a,** and stimulated with PMA/Ionomycin to determine cytokine expression. **(a)** Representative flow cytometry analysis of IL-17A^+^γδ T (CD45^+^CD3^+^γδTCR^+^IL-17A^+^, γδT17) cells. **(b)** Numbers of IL-17A^+^γδT17 cells isolated from skin-draining LNs. Pooled data from three independent experiments. Error bars represent SD. *P-values* were obtained by one-way ANOVA with Kruskal-Wallis-Test.
